# Optimizing Subretinal Bleb Formation for Visual Streak Involvement in a Porcine Model for Retinal Gene Therapy

**DOI:** 10.1167/iovs.65.12.41

**Published:** 2024-10-28

**Authors:** Immanuel P. Seitz, Tobias Peters, Felix Reichel, Andrea Bähr, Hanna Auch, Jan Motlik, Taras Ardan, Jana Juhasova, Yaroslav Nemesh, Stefan Juhás, Nikolai Klymiuk, Manuel Dominik Fischer

**Affiliations:** 1 University Eye Hospital, Eberhard-Karls-Universität Tübingen, Tübingen, Baden-Württemberg, Germany; 2 Oxford University Hospitals NHS Foundation Trust, Oxford Eye Hospital, Oxford, Oxfordshire, United Kingdom; 3 Medical Department I, Cardiology, Angiology, Pneumology, Klinikum rechts der Isar, Technische Universität München, München, Bayern, Germany; 4 Center for Innovative Medical Models, Ludwig-Maximilians-Universität München, München, Bayern, Germany; 5 Institute of Animal Physiology and Genetics, Akademie věd České republiky, Praha, Czechia; 6 Nuffield Department of Clinical Neurosciences, University of Oxford, The John Radcliffe Hospital, Oxford, United Kingdom

**Keywords:** gene therapy, cell therapy, subretinal injection, pig, porcine

## Abstract

**Purpose:**

Subretinal (SR) injection in porcine models is a promising avenue for preclinical evaluation of cell and gene therapies. Targeting of the subretinal fluid compartment (bleb) is critical to the procedure, especially if treatment of the cone-rich area centralis is required (i.e., visual streak [VS] in pigs). To our knowledge, this study is the first to investigate the influence of injection site placement on VS involvement in the pig eye.

**Methods:**

We performed 23-gauge pars plana vitrectomy followed by SR injection in 41 eyes of 21 animals (*Sus* *scrofa domesticus*). In 27 eyes (65.9%), the injection site was placed superior to the VS, and in 14 eyes (34.1%) it was placed inferior to it. Using intraoperative imaging, blebs were classified based on their propagation behavior relative to the VS.

**Results:**

In 79% of cases, blebs from inferior injection sites developed away from the VS, exhibiting a mean ± SEM vertical anisotropy (AP) of 0.67 ± 0.11. In contrast, blebs from superior injection sites tended to develop toward the VS with an AP of 1.27 ± 0.18 (*P* = 0.0070). Blebs developed away from the VS in only 41% of injections (*P* = 0.0212). Inferior blebs were orientated close to 0° (horizontal), whereas superior blebs displayed varied orientations with a mean angle of 56° (*P* = 0.0008).

**Conclusions:**

Bleb propagation was anisotropic (i.e., directionally biased) and dependent on injection site placement. Superior injection sites led to superior VS detachment. Morphological analysis suggested increased adhesion forces at the VS and superior vascular arcades. This study will aid the planning of surgeries for targeted subretinal delivery in pig models.

Pig models are a promising avenue of gene therapy research. The pig eye's overall dimensions^1^ are comparable to those of the human eye. Pigs also possess a cone-rich area centralis, called the visual streak (VS), which can serve as model for macular disease.^2^ The anatomical configuration of the visual streak is depicted in Figure 1. This is in contrast to murine models, which lack a dedicated area centralis.^3^ Furthermore, humanized transgenic pigs harboring genetic defects that cause inherited retinal diseases in humans have already been developed,^4^^,^^5^ which offers an advantage over other animal models such as non-human primates, which are anatomically more human like but still mostly available as wild types.^6^ Due to these benefits, pig models are increasingly being used to evaluate a range of research questions pertinent to gene and cell therapy,^7^ including the optimization of subretinal (SR) injection.^8^^,^^9^

**Figure 1.**
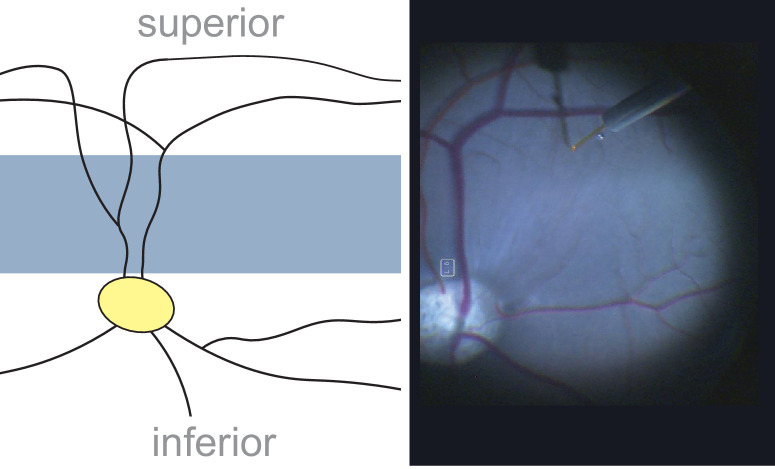
Anatomy of the central pig retina and area centralis. (**A**) Schematic drawing of the central pig retina. The VS (*blue area*), the cone-rich area centralis, is located just superior to the optic nerve head (*yellow oval*) and framed by the superior and inferior vascular arcades. The common trunk of the superior vascular arcades crosses the visual streak. (**B**) Corresponding intraoperative image showcasing the area centralis anatomy. Note the 41-gauge cannula primed for SR injection at a superior injection site.

SR injection remains the gold standard in gene therapy delivery, due to its high transduction efficiency in the outer retina and a favorable safety profile with regard to immune response,^10^^,^^11^ especially compared to intravitreal injection.^12^ In many gene and cell therapies that are applied using SR injection, there is a need to target a specific subset of cells, such as in the macula. Therefore, the success of SR injection often relies on the surgeon's ability to create a subretinal fluid compartment (bleb) that includes as much of the desired area as possible. To achieve this, it is necessary to account for the biomechanical forces in the subretinal space that guide the expansion of the infused volume. We hypothesized that one factor that guides subretinal bleb propagation in the pig eye is placement of the injection site. Following this hypothesis, this study compared two injection sites (superior vs. inferior to the visual streak) with regard to their capacity to detach the VS.

## Methods

### Subretinal Injections

**Figure 2.**
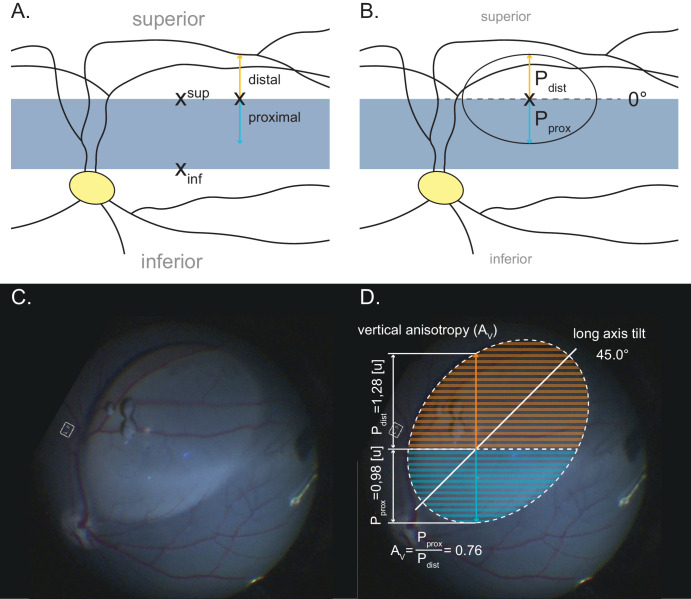
Injection site placement and bleb quantification. (**A**) Retinotomy site placement immediately superior and inferior to the visual streak. (**B**) Bleb quantification: The bleb base is approximated as an ellipse (continuous line). X denotes the retinotomy site. The distance between the retinotomy site and distal (P_dist_) and proximal (P_prox_) bleb margins is illustrated using colored arrows (P_prox_, *blue*; P_dist_, *orange*). For the depicted bleb, P_prox_ = P_dist_. The vertical propagation anisotropy (A_V_) is P_prox_/P_dist_ = 1; that is, there is no preference for either distal or proximal propagation of the subretinal fluid that is being injected. As the long (**A**) and short (**B**) axes of the ellipse (not marked) are sufficiently different in length (i.e., a/b > 1.1), the long-axis tilt (*dashed line*) can be measured. The long-axis tilt of the depicted bleb is 0° (i.e., temporal–nasal, or horizontal). (**C**) Image of a bleb post injection. (**D**) Bleb quantification measurements for the bleb depicted in **C**.

This study investigated subretinal bleb formation by analyzing surgical footage from gene therapy studies performed in pigs (*Sus* *scrofa domesticus*). All injections used the same surgical setting, equipment, surgeon, protocols, and consumables. The 23-gauge pars plana vitrectomy combined with subretinal injection was performed in 41 eyes of 21 animals. Animals were placed on an operating table and turned on their side. The head was positioned to ensure that the posterior pole of the treated eye was level to optimize surgical access and to minimize any influence of gravity. Then, 200 µL of vector solution or PBS was injected through an extendible 41-gauge subretinal injection needle (extendible 41 G subretinal injection needle, 23 gauge/0.6 mm; DORC, Zuidland, The Netherlands), using a foot-pedal–controlled injection system, similar to the clinical setting in humans. In 27 eyes (65.9%), the retinotomy site was placed immediately superior to the area centralis, and in 14 eyes (34.1%) it was placed immediately inferior to it (Fig. 2A). Borders of the visual streak were pre-identified during careful review of retinal imaging data of the individual animal just prior to surgery. Retinotomy sites were then placed after identifying the border of the visual streak by cross-referencing with landmarks of the retinal vasculature during surgery. Postoperatively, blebs were mapped by the surgeon using intraoperative camera footage. Quantification of blebs was performed in Illustrator 2024 (Adobe, San Jose, CA, USA) using the dimension tool and an area measurement script. All animals were of the same species and strain (PIGMOD midipigs) with no discernible difference in retinal anatomy or function at time of surgery. Animals were 3 months (±3 weeks) at the time of surgery. All procedures were conducted under the supervision of the responsible regulatory authorities. The State Veterinary Administration of the Czech Republic approved the subretinal intervention at IAPG Libechov under experimental protocol number 75/2019, and the procedures were compliant with the ARVO Statement for the Use of Animals in Ophthalmic and Vision Research.

### Vertical Propagation Anisotropy

Bleb shapes were characterized using several quantitative parameters, which determined if blebs displayed a tendency to evolve proximally (i.e., toward the VS) or distally (i.e., away from the VS). To quantify this, we measured the vertical distance P (P for propagation) between the retinotomy site and the distal (dist) and proximal (prox) bleb margins. The main derived readout was vertical propagation anisotropy (A_V_). It assigns a single value to the propagation behavior of each bleb relative to both the injection site and the visual streak (Figs. 2B, 2D). A_V_ was defined as P_prox_/P_dist_. For grouped analyses, blebs with an A_V_ of 0.9 to 1.1 were defined as isotropic (i.e., no clear tendency regarding proximal/distal propagation). Values > 1.1 indicated that blebs propagated toward the VS, and values < 0.9 indicated that they propagated away from the VS. The temporal–nasal component of bleb propagation was disregarded for this analysis, as it was irrelevant to the degree of VS involvement. To cross-validate the omission of the temporal–nasal component of bleb propagation, the analyses were repeated with area measurements. Here, the bleb areas proximal and distal to the injection site were measured instead of the vertical distance to the bleb margin (see hatched area in Fig. 2D).

### Long-Axis Tilt

Additional parameters included the long-axis (a) and short-axis (b) length of the bleb base (bleb bases were approximated as being elliptical in shape), as well as the angle at which the long axis was tilted (Fig. 2B). The grid axes for the tilt measurement were defined as follows: 0° temporal–nasal (horizontal) axis, 90° superior–inferior (vertical) axis. Long-axis tilt was only recorded for blebs that were sufficiently eccentric (i.e., non-circular). For this study a/b > 1.1 was determined to be sufficiently eccentric for a tilt measurement.

### Statistical Analysis

As the actual retinal distances in millimeters was not paramount to this research, distances were measured in arbitrary units. The statistical analysis was performed using Prism 10.1.1.270 for Macintosh (GraphPad, Boston, MA, USA). Descriptive statistics were used to summarize the data. Unpaired two-sided *t*-tests were used to compare the vertical anisotropy between the injection sites, as well as long-axis tilt. Continuous variables are given as mean ± SEM unless stated otherwise.

## Results

### Propagation Anisotropy and Injection-Site Placement

**Figure 3.**
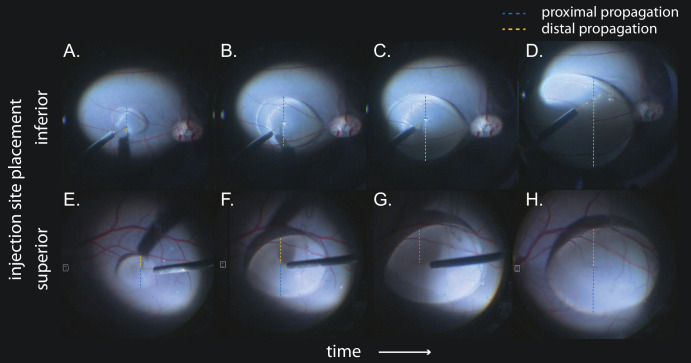
Sample bleb propagation over the course of injection by injection site. (**A**–**D**) Inferior injection site. The distance between the injection site and the distal and proximal bleb margins is marked with *dashed lines*. Distal bleb margin are indicated in *orange*; proximal bleb margin in *blue*. Early in the injection (**A**), immediately after the SR space was opened, the bleb was relatively balanced. Formally, the bleb was biased proximally (i.e., toward the visual streak). After injection of the first ∼50 µL (**B**), the bleb propagation balanced out. As more volume was injected (**C**, **D**), the bleb propagated increasingly away from the VS (final A_V_ = 0.35). (**E**, **F**) Superior injection site. Over the course of the injection, the bleb displayed a more balanced propagation. In the end, the bleb encompassed most of the VS (final A_V_ = 1.23).

Bleb propagation from the injection site was anisotropic (i.e., directionally biased) and dependent on injection-site placement. Blebs originating at the inferior injection site displayed a tendency to develop away from the area centralis (i.e., distally, or inferiorly) with an A_V_ of 0.67 ± 0.11. For blebs originating at superior injection sites, there was a tendency to develop toward the area centralis (i.e., proximally, also inferiorly) with A_V_ of 1.27 ± 0.18. Figure 2 gives two sample time sequences (one inferior, one superior) that illustrate bleb evolution over the course of injection. The difference in subretinal propagation behavior between injection sites was highly statistically significant (*P* = 0.0070) (Fig. 3B). The same result was found in cross-validation with bleb area measurements instead of vertical distance to bleb margin. The propagation anisotropy based on area measurements was 0.65 ± 0.50 for inferior injection sites and 1.41 ± 1.10 for superior injection sites (*P* = 0.0112).

### Superior Visual Streak Detachment With Superior Injection Sites

In a secondary analysis, blebs were grouped by their propagation behavior into three groups using their A_V_ values: toward VS (A_V_ > 1.1), away from VS (A_V_ < 0.9), or balanced (0.9 < A_V_ > 1.1). In summary, using an injection site immediately inferior to the VS resulted in 11 out of 14 blebs (78.6%) developing away from it. For superior retinotomy sites, this rate was significantly lower (*P* = 0.0212). Here, only 11 out of 27 blebs (40.7%) developed away from the VS.

### Bleb Tilt is Dependent on Injection Site

With regard to long-axis tilt, blebs originating from an inferior injection site were exclusively angled in a horizontal orientation (mean angle = 0°). The long axis of blebs originating from a superior retinotomy site displayed more varied angles, with a mean angle of 56° (Fig. 3B). The difference in long-axis orientation between injection sites was highly statistically significant (*P* = 0.0008).

## Discussion

The pig model is a promising large-animal model for the investigation of gene and cell therapies. As SR injection is still the gold standard in gene and cell therapy application, an increase of SR injections in pigs is to be expected. Targeting of select tissues such as the cone-rich area centralis (i.e., VS in pigs) is imperative for successful gene therapy studies. For the pig eye, no structured analyses are available to aid surgeons in targeting subretinal blebs to the cone-rich area centralis. To close this gap, this study aimed to quantitatively study the influence of retinotomy site placement on subretinal bleb propagation relative to the VS.

We hypothesized that blebs that form in isotropic conditions (i.e., equal resistance to detachment in every direction) should approximate a circular shape. This is not what we found in the pig SR space. SR propagation of blebs with regard to the injection site and VS was clearly anisotropic and highly dependent on injection site placement. Choosing a superior injection site resulted in a significant and meaningful improvement of VS involvement compared to an inferior approach. Blebs tended to develop proximally (i.e., toward the VS) for superior injection sites and distally (i.e., away from the VS) for inferior injection sites. Using an inferior retinotomy site resulted in a failure rate (bleb developing away) of 79%, compared to 41% for superior injection sites.

Furthermore, we hypothesized that directional propagation barriers can deform ellipsoid blebs into increasingly eccentric (i.e., squashed) shapes (long axis ≫ short axis). The finding that the bleb base long axis in inferior injection sites approximated 0° (horizontal) in all cases was in line with blebs being restricted by a horizontally aligned propagation barrier. Given that blebs tended to develop distally (i.e., inferiorly for inferior injection sites), this presumed barrier should be located above the inferior injection site. Taken together, our data on both bleb propagation and shape suggest that the VS acts as a relative detachment barrier compared to the surrounding retina, not dissimilar to the human fovea.

**Figure 4.**
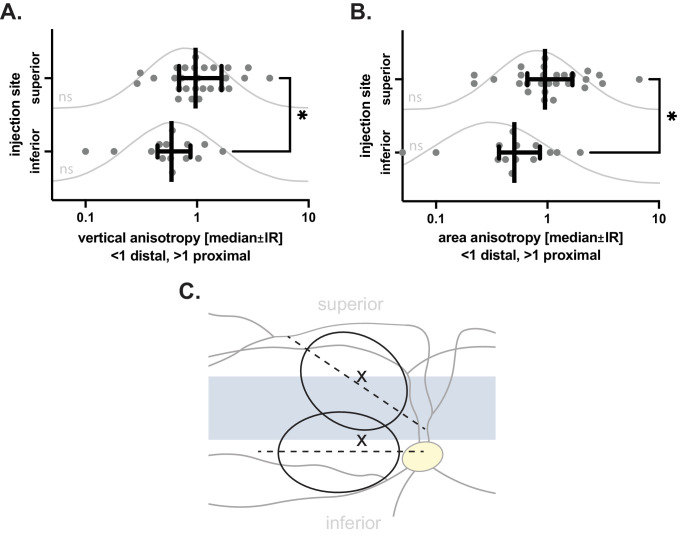
Average bleb morphology and vertical anisotropy by injection site. (**A**, **B**) Subretinal bleb propagation was anisotropic for both vertical anisotropy (**A**) and bleb area proximal versus distal of the VS (**B**). Placement of the injection site superior to the VS resulted in development of more balanced blebs, with a tendency to develop toward the VS. In contrast, placement inferior to the VS resulted in development of blebs away from the VS. This difference was highly statistically significant (A_V_, *P* = 0.0070; area, *P* = 0.0112). Lines and error bars represent median ± interquartile ranges. Curves represent lognormal fit. All values were distributed normally (D'Agostino–Pearson, *P* > 0.05). ns, not significant). (**C**) The average bleb morphology for both injection sites. Superior injection sites showed superior VS detachment (mean A_V_ = 1.27) compared to inferior injection sites (mean A_V_ = 0.67). The bleb long-axis tilt was in line with the visual streak and the common trunk of the superior vascular arcades, acting as detachment barriers (superior 55° vs. inferior 0°).

Regarding superior injection sites, the tendency of blebs to develop proximally (i.e., also inferiorly for superior retinotomy sites) suggests the presence of another detachment barrier superior to the superior injection site, which on average limited detachment even more than the VS. In addition, blebs originating from superior injection sites displayed more variation in their long-axis tilt, with a mean angle of 56°. This suggests that the presence of another, differently aligned detachment barrier confounded the (otherwise expected) horizontal alignment. Given the orientation of this tilt, this second barrier should be located superior or temporal to the injection site (compare Fig. 4A). The most likely candidate for this presumed detachment barrier is the common trunk of the superior vascular arcades, which originates at the optic nerve head and crosses the visual streak vertically before arching horizontally. The superior vasculature is on average more pronounced compared to the inferior arcades and could potentially provide more resistance. The more beneficial outcome when choosing superior injection sites could therefore be a consequence of the superior vascular arcades serving as a “counter-bearing” to the VS.

We see several limitations with this study. All surgeries were performed by a single surgeon. Although a core vitrectomy was performed, no staining (e.g., with triamcinolone) was performed to control for complete posterior vitreous detachment. Although the single-surgeon approach reduced inter-surgeon confounding factors across our sample, intra-surgeon factors such as handedness and individual surgical approach could not be controlled for. Regarding handedness, there was no significant difference between right and left eyes with regard to vertical propagation anisotropy (*P* = 0.19) and bleb area (*P* = 0.20). Future studies should incorporate more surgeons or make use of a robotic injection systems to address these concerns. Of note, a large study that investigated the directional bias of bleb propagation in human gene therapy patients found that blebs do not respect subretinal cannula direction (i.e., the three-dimensional angle from which the cannula entered the subretinal space). The only significant factors that determined directional bias of blebs were patients’ age and the underlying retinal disease.^13^ This makes it somewhat unlikely that handedness plays a critical role in bleb propagation. In addition, there was no indication that viscosity of the injectate modified the results, as there was no difference in propagation between blebs formed via injection of adeno-associated virus versus PBS (*P* = 0.57).

Finally, although bleb propagation is itself an immediately relevant endpoint for surgeons, it can only be a surrogate for the forces acting in the subretinal space. This means that the abovementioned hypotheses about the adhesion behavior of different anatomical structures do not preclude the results of direct mechanical measurements. The same goes for intraoperative volumetric data, which could provide additional information. Unfortunately, volumetric data were not available, as intraoperative optical coherence tomography (OCT) could not be performed in this study. Postoperative OCT scans were only performed weeks after the surgery as part of a larger gene therapy study, but these scans were not very useful to addressing the questions posed in this manuscript as they do not show the blebs (baseline before surgery, follow-up weeks after bleb re-absorption). Fortunately, evidence suggests that (in the absence of an air tamponade) the bleb remains very localized, suggesting that quantification of the bleb area is a valid surrogate for treated area.^14^ In summary, this study provides a powerful yet easy to follow guideline for vitreoretinal surgeons to meaningfully optimize subretinal treatment with gene and cell therapies in the pig eye.
